# STEM education needs for human movement sciences professionals

**DOI:** 10.3389/fneur.2024.1503022

**Published:** 2025-01-10

**Authors:** Leonardo Gizzi, Francesco Felici

**Affiliations:** ^1^Department of Biomechatronics, Fraunhofer Institute for Manufacturing Engineering and Automation, Stuttgart, Germany; ^2^Department of Movement, Human, and Health Sciences, Laboratory of Exercise Physiology, University of Rome “Foro Italico”, Rome, Italy

**Keywords:** STEM, education, Sport Sciences, electromyography, neuromechanics, neurophysiology

## 1 Introduction

STEM disciplines are fundamental tools for a quantitative and reliable understanding of physical phenomena. Despite that, it often happens that students start their academic journey with scarce information on these topics, and sometimes lack the basic concepts for understanding even relatively straight-forward tools like reading graphs, or interpreting data based on their distribution. Large Language Models and, in general, Artificial Intelligence are revolutionizing the way humans interact with computers and promise to democratize data analysis also for non-tech-saviors (1)—prompt writing being the key to unlock their potential (2). As for any data manipulation activity, however, the quality of the outputs is nothing more than the reflection of the quality of the inputs (in this case, not only the data, but the very way the data manipulation is requested). Without a proper understanding of the fundamental concepts of STEM (Science Technology Engineering And Mathematics) and a formal education on prompting we face the risk of an invasion of largely perfectible outputs that will inevitably (and irreparably) “poison the well” for the outputs to come. In this opinion paper we discuss the case of a hypothetical surface electromyography (sEMG) academic course, as an example of a STEM-based, multi-disciplinary topic that could appeal students and involve teachers from multiple disciplines. The choice of sEMG was dictated not only by our professional understanding of the discipline, but by its growing clinical relevance (3) and overall diffusion. We believe that such a Syllabus may provide an easily implementable step toward a stronger STEM-oriented and evidence-based approach in several disciplines—like, for example, Physiotherapy (4) or Sport Sciences. This Opinion Paper only proposes a syllabus for a sEMG course and does not address the important issue of pedagogical methodology, that would require an extensive discussion once an agreement has been reached on the topics that should be taught. Although it is not unheard of that neurophysiological topics can be taught with minimal STEM-related knowledge [see for example (5, 6)], it is our strong opinion that the latter provides a much better understanding of the phenomena and, as a consequence, a far deeper usability of the measurement results. Finally, it should be considered that the contents of the current example can be easily complemented or extended to reach out to other disciplines, like quantum-based neuromagnetic sensing (7).

## 2 Why a syllabus?

It is uncontroversial that advancement in sEMG technique of the last 10 years coupled to the increased power of the analytical tools specifically designed to decipher the sEMG (8)—High Density sEMG (HDsEMG)—opened an important window for a non-invasive investigation of the central nervous system (CNS) motor strategies in healthy, stroke (9), and spinal cord injured (SCI) subjects. Moreover, electromyographic activity is routinely used as a mean to control assistive and rehabilitation tools such as prostheses, exoskeletons and visual feedback devices. These developments not only moved the sEMG field into the area of medical imaging but also provided tools to investigate the neural drive to a muscle (10) and motor coordination.

These techniques are still relatively novel, therefore, time is needed until their clinical utility is widely accepted and their use is widespread. Specialized, interdisciplinary courses across universities and clinical environments are needed to train and teach the future generations of sport and exercise physiologists, physiotherapists and clinicians.

Almost 30 years ago, De Luca (11) warned that “sEMG is a seductive muse” because it may seem relatively easy to harvest myoelectric signals from a pair of electrodes placed over the skin, whilst a comprehensive interpretation of the underlying phenomena that they represent would require compound knowledge in several disciplines (such as, but not limited to, signal processing and neuromechanics). It seems that such an underestimation of the complex processes behind sEMG signal analysis is still among us, while understanding and interpreting sEMG, from simplistic to more sophisticated approaches, requires an extensive and multidisciplinary background that appears to be lacking today. As a matter of fact, we (12) have already reported that only a fraction (5% of the total) of the top 100 university (extracted via Quacquarelli Symonds World Ranking and Shanghai Ranking) focused on Human Movement, Biomechanics, Sport Science, Physiotherapy and Exercise Physiology, educate students on the fundamental principles for studying the neural control of muscles at the direct motor unit level. Another study (13) reported that, although over 83% of the respondents to a survey considered sEMG modereately to very important for their research only a mere 3.2% were using it in their practice. This was attributed to a lack of undergraduate preparation (that did not improve, in the majority of cases, after graduation). Thus, there is critical need to open the access to these technologies and to instruct teachers (long before students) across the disciplines of these fundamentals. Although the physiological and engineering knowledge is mature to routinely monitor the spinal cord output by non-invasive high-density sEMG recordings, we are failing to generalize and make them fully available to the current generation of students. It must be stressed that at least education of sport professionals is widely left to the “wild”: academic, well structured, courses are challenged by so many different non-academic actors often based on the field experience of (more or less) talented individuals rather than on scientific evidence. This is, of course, a political problem with strong economic implications, not relevant for the present opinion that should, however, be kept in mind.

An extensive literature, from textbooks, journals and websites, addresses the barriers limiting the widespread use of sEMG. There is general agreement that the most important barrier is the lack of education in the field (12, 14–18). Despite the availability of textbooks (19), open access books,^1^ online teaching material,^2^ and the efforts of the International Society for Electrophysiology and Kinesiology^3^ in the preparation of Tutorials (10, 20–25), and Consensus Papers (20, 26–30), academic courses on sEMG, for medical doctors, Health Sciences Exercise/Sport degrees, are extremely rare. Therefore, it seems useful to propose a syllabus for one. For disclaimer, however, we are aware that such courses may already exist (e.g., at Imperial College,^4^ University of Stuttgart,^5^ the Free University of Amsterdam,^6^ or the Politechnical University of Torino^7^—just to name a few), but their adoption is far less ubiquitous than we believe it should be.

## 3 A proposal for a syllabus of a course on sEMG

In this section, we indicate the contents of a hypothetical, interdisciplinary academic course on neuromechanics that, we believe, could bridge the gap between the current distribution of knowledge. We would expect that the students emerging from such a course would be able to independently conduct measurements and interpret the results at a non-medical level (on the other hand, for an ubiquitous adoption of sEMG-based diagnostics, also clinicians should receive some formal training at least in how to interpret the data). l. Since muscles act as biological actuators of the central nervous system commands, their mechanical and electrical (sEMG) outputs are causally associated and are a manifestation of the corticospinal volley to the muscle. Therefore, an understanding of neuromechanics (i.e., biomechanics and motor control principles) requires a background in physics and electrophysiology. These may be topics of other courses that should be coordinated. The middle- and high-school curricula of most countries (12 years) provide basic concepts in math and physics. Many of these concepts—necessary to read and understand the basic literature on sEMG and neuromechanics—should therefore be already available when accessing academic courses. However, it often appears necessary to refresh at least a few of them and point out materials or basic topics to review (Table 1). The basic knowledge of these concepts should be a tested prerequisite for admission to a sEMG course; without such tests, the risk is that not even a common alphabet of logic instruments could be established. Items 1–4 in Table 1 describe topics that must be refreshed, using previously available material and textbooks, or—for example—freely available online material such as modules 1, 2, and 3, of “robertomerletti.it”.^8^ More novel sEMG-focused material is listed in items 5 and 6 in Table 1 and in modules 4–10 of the above link. All the concepts listed in this material are required for understanding the sEMG literature.

**Table 1:** Proposed syllabus for a course on basic concepts needed in biomechanics and in surface EMG electrophysiology.

	**Basic mathematics and neuromechanics**		
1 Refreshing basic mathematics, algebra, geometry, trigonometry, calculus, and statistics	Powers and logarithms Basic algebra and trigonometry Basic statistics (mean and standard deviation at College level)	2 Refreshing basic physics and biomechanics measurements and international units	Physical quantities Concepts of linear and angular displacement, velocity, and acceleration Instantaneous and average values
	Basic geometry, 2D and 3D linear, polar and logarithmic coordinate systems, scalars and vectors		Forces and their lever arms Levers and pulleys Concept of torque. Concept of inertia and of moment of inertia. Newton's law Balance of forces and torques Mechanical energy and power
	Vector arithmetic, composing and decomposing vectors Concept of matrix and basic matrix operations		
	Plot of a variable versus another. Cartesian and polar plots		Goniometers, accelerometers, gyroscopes, magnetometers, IMU sensors Biomechanical gait analysis. Stereophotogrammetry and joint angles Load cells and force plates
	Concept of functions in 1-D and 2-D. Basics of differentiation and integration of a function Maxima and minima of a function Basics of programming in MATLAB and/or Python		
	Basic statistical tests The concept of statistical significance		Movements of the human joints Forces and torques at the human joints. Ground reaction force and its distribution under the feet and in time Muscle forces. Kinematics and dynamics (kinetics) of movements. Concentric and eccentric contractions
	**Basic electrophysiology and surface electromyography (sEMG)**		
3 Refreshing basic electrical physics quantities	Basic concepts of charge, voltage, current and resistance in DC Ohm's law. The electrical-hydraulic analogy The voltage divider. Batteries and the power line. The sinusoid and its properties. Sinusoidal voltages and currents Concept of phase. Concept of impedance. Concept of waveform	4 Refreshing basic electro-physiology of sEMG	The neuron and the muscle fibres, the innervation zone (IZ) and the motor unit (MU) The single fiber and the MU action potentials (MUAP) Generation, propagation and extinction of MUAPs. The end-of-fiber effect MU recruitment strategies and discharge rates. Henneman's principle Summing force twitches and MUAP trains The sEMG as a sum of MUAP trains and as a random signal. The sEMG as a movie. Instantaneous sEMG voltage maps
	The concepts of capacitance and its impedance. Parasitic capacitances with the power line and their effects on sEMG		Differences between needle, thin-wire and sEMG. Sampling the map in space in one point (single channel monopolar signal) and in many points (HDsEMG). Grids of electrodes. Small and large pixels
	The electrode-electrolyte junction. The electrode-skin impedance and noise The input impedance of an amplifier		Surface extension of a MUAP. Crosstalk and end of fiber effect. Definition of muscle fiber conduction velocity. Effect of electrode location with respect to the IZ Pinnate muscles and pinnation types
5 Fundamental concepts of biomedical signal detection and processing	The concept of signal and noise and their spectra Periodic and random-like signals. Signals in space and time, 1D and 2D signals. Music, pictures, and movies. Stationarity of a signal	6 Acquisition of sEMG. understanding and interpretation of its features	Monopolar and differential detection HDsEMG detection. Spatial filters. Muscle fibre conduction velocity. Artifacts and power line interference: how to avoid or remove them. Features of the front-end amplifier: CMRR and input impedance. Gain. Signal conditioning and analog filtering
	A periodic signal as a sum of sinusoids (harmonics) The Fourier series		Amplitude estimators. Fourier analysis of the sEMG. The amplitude and power spectra of the sEMG. Estimation interval (time epoch), smoothing of the estimates.
	A random signal as a sum of sinusoids (harmonics) The Fourier transform		Myoelectric manifestations of muscle fatigue. What are the sEMG amplitude and spectral estimates telling us?
	Concepts of bandwidth, amplitude, phase and power spectra of a signal. Examples from audio spectrum analysers		Decomposition of the HDsEMG signal into the constituent MUAP trains. Identification of single MU properties The concept of neural drive
	Amplitude (ARV and RMS) of a signal. Envelope of a signal		Multichannel sEMG and muscle coordination. Estimating load sharing
	The concept of frequency content and of filtering of a signal in time and space. Filtering of a music piece and of an image		Mathematical models of sEMG and their applications in signal interpretation. Modelling the effect of specifics physiological/anatomical/geometrical changes. Limitations of models
	Binary numbers Conversion of a signal into a series of binary number. Sampling and the Nyquist rate. A/D conversion, A/D converters		Applications in biofeedback, monitoring of neuromuscular changes, and assessment of effectiveness of interventions. Stroke, Parkinson, ALS, spasticity, gait and movement analysis
	The main bioelectric signals and their clinical relevance: ECG, EEG, needle and thin wire EMG, surface EMG (sEMG)		Advanced applications in neural engineering. AI and sEMG. Control of artificial limbs, orthosis, and exoskeletons. Discussion of papers and applications. Future perspectives
Useful URLs, teaching material, and textbooks (see references for articles): 1. ISEK and JEK tutorials and papers on Consensus for Experimental Design in Electromyography (CEDE Project) (https://www.isek.org), 2. Surface Electromyography for Non-Invasive Assessment of Muscles (EU Project SENIAM, 1999) (https://www.seniam.org) 3. Teaching material (clinical and technical) in https://www.robertomerletti.it (10 free teaching modules with over 500 slides 30 videos and some technical notes) 4. Teaching material prepared within the EU Project: https://Teacherasmusplus.eu/news and https://teach.ibv.org			
Recommended textbooks: 1. McCaw S. *Biomechanics for Dummies*. Hoboke, NJ: J. Wiley (2014). ISBN 978-11-118-67469-7 2. Knutson D. *Fundamentals of Biomechanics*. New York, NY: Springer (2007). ISBN 978-0-387-49311-4 3. Barbero M., Rainoldi A, Merletti R. *Atlas of Muscle Innervation Zones: Understanding Surface EMG and its Applications*. Milano: Springer (2012). ISBN 978-88-470-2462-5 4. Merletti R, Farina D. (editors) Surface Electromyography: physiology, engineering and applications, IEEE Press/J Wiley, USA, 2016, ISBN: 978-1-118-98702-5 (for advanced students) 5. Merletti R, Campanini I, Rymer WZ, Disselhorst-Klug C (editors). *Surface electromyography: barriers limiting widespread use of sEMG in clinical assessment and neurorehabilitation*. Open Access e-book. Frontiers in neurology/Neurorehabilitation (2021). https://www.frontiersin.org/research-topics/11157/ 6. Samani A. *An Introduction to Signal Processing for Non-engineers*. Boca Raton, FL: CRC Press (2020). ISBN 13:978-0-367-20755-7 (for advanced students)			
Recommended (minimal) frontal lecture duration:			
Refreshing basic mathematics and biomechanics 1. Basic mathematics algebra, geometry, trigonometry, calculus, and statistics: ~6 h 2. Basic physics and biomechanics. Measurements and international units: ~5 h			
Basic electrophysiology and surface electromyography (sEMG) 3. Basic electrical physical quantities: ~6 h 4. Basic electrophysiology of sEMG ~3 h 5. Fundamental concepts of biomedical signal detection and processing ~5 h 6. Acquisition of sEMG. Understanding and interpretation of its features ~5 h 7. Discussion of papers and applications ~5 h Total Lecture time: ~35 h Laboratory demonstrations and practice: ~35 h			
Teaching relies extensively on the URLs, websites, and sections of textbooks listed above			

### 3.1 Fundamental concepts of signal processing and interpretation of the sEMG and its features

Items 5 and 6 would be the heart of the course and are very likely totally new to a sEMG beginner (Table 1).

Item 5 introduces the concepts of amplitude and envelope of a signal—of paramount importance, for example, in sEMG biofeedback, for the definition of muscle activation timing and intervals, and (with caution) for the estimation of exerted force. Item 5 also defines periodic and random signals and noise in time and space (e.g., skin surface) and the concept of Fourier transform and spectral content. These are the foundations of the concepts of (a) filtering, (b) myoelectric manifestations of muscle fatigue and, (c) EEG-EMG and EMG-EMG coherence. The technical concepts of bandwidth of a signal, sampling, and A/D conversion are important for defining the performance and the choice of an acquisition system. This stands valid for the analysis of all bioelectric signals (electrocardiography—ECG, electroencephalography—EEG, EMG).

Item 6 specifically focuses on sEMG detection and analysis and deserves some attention. Detection of sEMG implies an electrode “montage” which can be monopolar, bipolar (or single differential) which approximates the derivative of the signal, double differential, Laplacian, or an electrode grid which approximates the sEMG “image” on the skin. Electrode size and interelectrode distance affect the sEMG waveshape and its Fourier spectrum because they introduce a spatial filter (25, 26) The location of an electrode pair with respect to the muscle innervation zone is a very important factor affecting sEMG amplitude and spectral features (19).

Different detection modalities and electrode parameters make the comparison sEMG recordings non trivial, for reasons that may not be immediately intuitive and that must be understood. The quality of skin preparation and electrode-skin interfaces have a strong impact on the artifacts and power line interference as well as the input impedance (not just resistance!) of the front-end amplifier. The definition of muscle activation intervals may be quite dependent on the algorithm used. Notch filters to remove power line interference should be used with caution and possibly avoided. Muscle fiber conduction velocity affects the sEMG power spectrum and can be estimated using properly placed electrode arrays and suitable algorithms; it is an important physiological parameter that determines myoelectric manifestations of muscle fatigue as well as muscle fiber membrane alterations.

The sEMG activity patterns for individual muscles (e.g. in the gait cycle) exhibit a great deal of intersubject, intermuscular and context-dependent variability. The latter seems to be obtained by different combinations of a few (< 10) elementary patterns or modules by the CNS (31).

Mathematical modeling of a muscle is an important teaching tool and provides (with caution due to its limitations and approximations) a way to solve the “inverse problems” of estimating features of the muscle given its sEMG (mostly HDsEMG). With the help of artificial intelligence (AI), modeling is leading to the creation of “digital twins” [Maksymenko2023] with a potential to greatly affect clinical practice.

The algorithms for the decomposition of sEMG into the constituent motor unit action potantial (MUAP) trains created new tools for the non-invasive estimation of (a) the neural drive to the muscle and (b) the order of recruitment and de-recruitment of MUs. Their application requires the use of HDsEMG and provides a “measurement” of the spinal cord output to one or more muscles as well as the degree of “coherence” between the neural drive to different muscles; it is one of the foundations of the new field of Neural Engineering.

## 4 Conclusion

Despite the considerable number of technical papers (>15,000), the significant numbers of clinical applications—(32–39) among many others—the few articles discussing education (12, 14–17, 40), the efforts of the European Community (Projects SENIAM^9^ and TEACH^10^) and of ISEK (sEMG Tutorials and Consensus Papers), and the availability of textbooks and online teaching material, sEMG is not yet a teaching subject in almost all academic curricula for rehabilitation MDs, physiotherapists and movement scientists.

An explanation often given for this anomaly is that there are insufficient clinical studies demonstrating the validity of sEMG as an assessment tool and no accepted protocols for its application. While this is partially true, it must be recognized that clinical studies and protocols require access to patients and must be planned by clinicians. But very few clinicians have sufficient knowledge of the field to prepare proposals (14). In private practice, assessment of effectiveness of applied treatments is certainly not rewarding. Furthermore, sEMG is considered a “difficult” field for which the entry level of freshmen is considered insufficient and impossible to integrate because of lack of time.

The physiotherapy and kinesiology professions are based on patient's feedback, rarely on measurements. Frequent remarks and questions are: (a) equipment is expensive, (b) why should I measure muscles? The PT and kinesiology professions are based on patient's feedback, not on measurements, (c) how am I supposed to use the results of sEMG measurements? How should they affect my decisions? (d) patient feedback is more than sufficient: there is no need for measurements,..., etc. It is obvious that the gap is cultural and that these professions are unprepared to the coming impact of AI (41, 42).

It is hoped that providing and describing the syllabus of a sEMG course indicating the need to refresh basic mathematics and physics (mostly online) can remove some of the difficulties pointed out in this field.

However, the problem originates far away from Academia. As mentioned in the book “The power of habit” (43), the infant mortality rate in the USA in the 60s was lowered by reducing the malnourishment of the future potential mothers through a better education of their high school teachers in the fields of nutrition science and biology. Similarly, movement and sport scientists and physiotherapists, dealing with people of any age, health, and fitness levels, may contribute to a healthier population and reduce health-related costs (and the consequences of neuromuscular disorders) through a better education and training of their teachers in the fields of measurements and Evidence-Based Practice. Suitable Train-The-Trainers (TTT) pedagogical models should be investigated for this purpose (44). Sport scientists—an example that is closest to our personal and professional experience—are not only employed in training high-level athletes, that move millions in sponsorships and advertisement; most of them work in close contact with individuals of every age and fitness levels and contribute to a healthier population. At the risk of sounding exaggerated, we believe that a decisive intervention on the way STEM disciplines are perceived and taught, in high-school as in the Universities, may radically improve the quality of life of millions of people, reduce health-related costs and promote longer and healthier lives.

## Footnotes

^1^https://www.frontiersin.org/research-topics/5033/; https://www.frontiersin.org/research-topics/11157/; https://doi.org/10.3390/s22207966; https://www.mdpi.com/books/reprint/7130

^2^ https://www.robertomerletti.it/

^3^ https://www.isek.org

^4^ https://www.imperial.ac.uk/neuromechanics-rehabilitation-technology/

^5^ https://www.imsb.uni-stuttgart.de/teaching/

^6^https://research.vu.nl/en/courses/electromyography-4; https://vu.nl/en/education/phd-courses/electromyography-1st-year

^7^ https://en.unito.it/ugov/degreecourse/1332817

^8^ https://www.robertomerletti.it/en/emg/material/teaching/

^9^ https://www.seniam.org

^10^ https://teach.ibv.org/

## References

[1] Sv S Sunil S AS PA Satish G. Democratizing data science: using language models for intuitive data insights and visualizations. In: 2024 4th International Conference on Pervasive Computing and Social Networking (ICPCSN). Salem: IEEE (2024), p. 1065–9. 10.1109/ICPCSN62568.2024.00177

[2] Giray L. Prompt engineering with ChatGPT: a guide for academic writers. Ann Biomed Eng. (2023) 51:2629–33. 10.1007/s10439-023-03272-4 37284994

[3] Campanini I Disselhorst-Klug C Rymer WZ Merletti R. Surface EMG in clinical assessment and neurorehabilitation: barriers limiting its use. Front Neurol. (2020) 11:934. 10.3389/fneur.2020.00934 32982942 PMC7492208

[4] Scurlock-Evans L Upton P Upton D. Evidence-based practice in physiotherapy: a systematic review of barriers, enablers and interventions. Physiotherapy. (2014) 100:208–19. 10.1016/j.physio.2014.03.001 24780633

[5] Lennartz RC. Electrophysiology of the undergraduate neuroscience student: a laboratory exercise in human electromyography. Adv Physiol Educ. (1999) 277:S42. 10.1152/advances.1999.277.6.S42 10644259

[6] Mathew AJ Nundy M Chandrashekaran N Oommen V. Wrestle while you learn: EMG as a teaching tool for undergraduate skeletal muscle physiology teaching. Adv Physiol Educ. (2019) 43:467–71. 10.1152/advan.00029.2019 31460776

[7] Gizzi L Marquetand J Köllner J Rölver R Jaglauber K Siegel M. Nitrogen-vacancy centers for prosthesis control. In: Biophotonics in Exercise Science, Sports Medicine, Health Monitoring Technologies, and Wearables, Vol. 12838. San Francisco, CA: SPIE (2024), p. 92–101. 10.1117/12.3022607

[8] Farina D Holobar A. Characterization of human motor units from surface EMG decomposition. Proc IEEE. (2016) 104:353–73. 10.1109/JPROC.2015.2498665

[9] Gizzi L Nielsen JF Felici F Ivanenko YP Farina D. Impulses of activation but not motor modules are preserved in the locomotion of subacute stroke patients. J Neurophysiol. (2011) 106:202–10. 10.1152/jn.00727.2010 21511705

[10] Del Vecchio A Holobar A Falla D Felici F Enoka R Farina D. Tutorial: analysis of motor unit discharge characteristics from high-density surface EMG signals. J Electromyogr Kinesiol. (2020) 53:102426. 10.1016/j.jelekin.2020.102426 32438235

[11] De Luca CJ. The use of surface electromyography in biomechanics. J Appl Biomech. (1997) 13:135–63. 10.1123/jab.13.2.135

[12] Felici F Del Vecchio A. Surface electromyography: what limits its use in exercise and sport physiology? Front Neurol. (2020) 11:578504. 10.3389/fneur.2020.578504 33240204 PMC7677519

[13] Bertoni G Leuzzi G Job M De Simone M Testa M. Exploring knowledge, perception, and use of surface electromyography in physiotherapy post graduate trainees in Italy: a single center preliminary survey. Front Rehabil Sci. (2024) 5:1489927. 10.3389/fresc.2024.1489927 39502556 PMC11534621

[14] Merletti R. Metrology in sEMG and movement analysis: the need for training new figures in clinical rehabilitation. Front Rehabil Sci. (2024) 5:1353374. 10.3389/fresc.2024.1353374 38348456 PMC10859507

[15] Campanini I Merlo A Disselhorst-Klug C Mesin L Muceli S Merletti R. Fundamental concepts of bipolar and high-Density surface EMG understanding and teaching for clinical, occupational, and sport applications: origin, detection, and main errors. Sensors. (2022) 22:4150. 10.3390/s22114150 35684769 PMC9185290

[16] Merletti R Temporiti F Gatti R Gupta S Sandrini G Serrao M. Translation of surface electromyography to clinical and motor rehabilitation applications: the need for new clinical figures. Transl Neurosci. (2023) 14:20220279. 10.1515/tnsci-2022-0279 36941919 PMC10024349

[17] Jette AM. Overcoming ignorance and ineptitude in 21st century rehabilitation. Phys Ther. (2017) 97:497–8. 10.1093/ptj/pzx037 28605558

[18] Merletti R Campanini I Rymer WZ Disselhorst-Klug C. Surface electromyography: barriers limiting widespread use of sEMG in clinical assessment and neurorehabilitation. Front Neurol. (2021) 12:642257. 10.3389/fneur.2021.642257 33643215 PMC7906963

[19] Barbero M Merletti R Rainoldi A. Atlas of Muscle Innervation Zones: Understanding Surface Electromyography and its Applications. Cham: Springer Science & Business Media (2012). 10.1007/978-88-470-2463-2

[20] McManus L Lowery M Merletti R. Søgaard K, Besomi M, Clancy EA, et al. Consensus for experimental design in electromyography (CEDE) project: terminology matrix. J Electromyogr Kinesiol. (2021) 59:102565. 10.1016/j.jelekin.2021.102565 34102383

[21] Avrillon S Hug F Baker SN Gibbs C Farina D. Tutorial on MUedit: an open-source software for identifying and analysing the discharge timing of motor units from electromyographic signals. J Electromyogr Kinesiol. (2024) 77:102886. 10.1016/j.jelekin.2024.102886 38761514

[22] Valli G Ritsche P Casolo A Negro F De Vito G. Tutorial: analysis of central and peripheral motor unit properties from decomposed high-density surface EMG signals with openhdemg. J Electromyogr Kinesiol. (2023) 74:102850. 10.1016/j.jelekin.2023.102850 38065045

[23] Clancy EA Morin EL Hajian G Merletti R. Tutorial. Surface electromyogram (sEMG) amplitude estimation: best practices. J Electromyogr Kinesiol. (2023) 72:102807. 10.1016/j.jelekin.2023.102807 37552918

[24] Merletti R Cerone G. Tutorial. Surface EMG detection, conditioning and pre-processing: best practices. J Electromyogr Kinesiol. (2020) 54:102440. 10.1016/j.jelekin.2020.102440 32763743

[25] Merletti R Muceli S. Tutorial. Surface EMG detection in space and time: best practices. J Electromyogr Kinesiol. (2019) 49:102363. 10.1016/j.jelekin.2019.102363 31665683

[26] Besomi M Hodges PW Van Dieën J Carson RG Clancy EA Disselhorst-Klug C. Consensus for experimental design in electromyography (CEDE) project: electrode selection matrix. J Electromyogr Kinesiol. (2019) 48:128–44. 10.1016/j.jelekin.2019.07.008 31352156

[27] Besomi M Hodges PW Clancy EA Van Dieën J Hug F Lowery M. Consensus for experimental design in electromyography (CEDE) project: amplitude normalization matrix. J Electromyogr Kinesiol. (2020) 53:102438. 10.1016/j.jelekin.2020.102438 32569878

[28] Gallina A Disselhorst-Klug C Farina D Merletti R Besomi M Holobar A. Consensus for experimental design in electromyography (CEDE) project: high-density surface electromyography matrix. J Electromyogr Kinesiol. (2022) 64:102656. 10.1016/j.jelekin.2022.102656 35344841

[29] Besomi M Devecchi V Falla D McGill K Kiernan MC Merletti R. Consensus for experimental design in electromyography (CEDE) project: checklist for reporting and critically appraising studies using EMG (CEDE-Check). J Electromyogr Kinesiol. (2024) 76:102874. 10.1016/j.jelekin.2024.102874 38547715

[30] Dick TJ Tucker K Hug F Besomi M van Dieën JH Enoka RM. Consensus for experimental design in electromyography (CEDE) project: application of EMG to estimate muscle force. J Electromyogr Kinesiol. (2024) 79:102910. 10.1016/j.jelekin.2024.102910 39069427

[31] Ivanenko YP Poppele RE Lacquaniti F. Spinal cord maps of spatiotemporal alpha-motoneuron activation in humans walking at different speeds. J Neurophysiol. (2006) 95:602–18. 10.1152/jn.00767.2005 16282202

[32] Merlo A Bo MC Campanini I. Electrode size and placement for surface EMG bipolar detection from the brachioradialis muscle: a scoping review. Sensors. (2021) 21:7322. 10.3390/s21217322 34770627 PMC8587451

[33] Rasool G Afsharipour B Suresh NL Rymer WZ. Spatial analysis of multichannel surface EMG in hemiplegic stroke. IEEE Trans Neural Syst Rehabil Eng. (2017) 25:1802–11. 10.1109/TNSRE.2017.2682298 28320672 PMC6492268

[34] Felici F. Neuromuscular responses to exercise investigated through surface EMG. J Electromyogr Kinesiol. (2006) 16:578–85. 10.1016/j.jelekin.2006.08.002 17052919

[35] Minetto MA Holobar A Botter A Ravenni R Farina D. Mechanisms of cramp contractions: peripheral or central generation? J Physiol. (2011) 589:5759–73. 10.1113/jphysiol.2011.212332 21969448 PMC3249048

[36] Zwarts MJ Stegeman DF. Multichannel surface EMG: basic aspects and clinical utility. Muscle Nerve. (2003) 28:1–17. 10.1002/mus.10358 12811768

[37] Drost G Stegeman DF van Engelen BG Zwarts MJ. Clinical applications of high-density surface EMG: a systematic review. J Electromyogr Kinesiol. (2006) 16:586–602. 10.1016/j.jelekin.2006.09.005 17085302

[38] Drost G Verrips A van Engelen BG Stegeman DF Zwarts MJ. Involuntary painful muscle contractions in Satoyoshi syndrome: a surface electromyographic study. Mov Disord. (2006) 21:2015–8. 10.1002/mds.21088 16972238

[39] Bhadane M Liu J Rymer WZ Zhou P Li S. Re-evaluation of EMG-torque relation in chronic stroke using linear electrode array EMG recordings. Sci Rep. (2016) 6:28957. 10.1038/srep28957 27349938 PMC4923947

[40] Trumbower RD Wolf SL. A forward move: interfacing biotechnology and physical therapy in and out of the classroom. Phys Ther. (2019) 99:519–25. 10.1093/ptj/pzz008 30690519 PMC7325447

[41] Rowe M Nicholls DA Shaw J. How to replace a physiotherapist: artificial intelligence and the redistribution of expertise. Physiother Theory Pract. (2022) 38:2275–83. 10.1080/09593985.2021.1934924 34081573

[42] Rowe M Osadnik CR Pritchard S Maloney S. These may not be the courses you are seeking: a systematic review of open online courses in health professions education. BMC Med Educ. (2019) 19:1–11. 10.1186/s12909-019-1774-9 31521150 PMC6744630

[43] Duhigg C. The Power of Habit: Why We do What We do and How to Change. New York, NY: Random House. (2013).

[44] Pearce J Mann MK Jones C Van Buschbach S Olff M Bisson JI. The most effective way of delivering a train-the-trainers program: a systematic review. J Contin Educ Health Prof . (2012) 32:215–26. 10.1002/chp.21148 23173243

